# Functional Characteristics of Fungal Communities in the Rhizosphere of the Endangered Plant *Abies ziyuanensis*

**DOI:** 10.3390/microorganisms13091989

**Published:** 2025-08-26

**Authors:** Yufeng Wang, Jiahao Wu, Tao Deng, Jiatong Ye, Xinghua Hu

**Affiliations:** 1 Guangxi Key Laboratory of Plant Functional Phytochemicals and Sustainable Utilization, Guangxi Institute of Botany, Chinese Academy of Sciences, Guilin 541006, China; wyf03170517@163.com (Y.W.); 1612967690wu@gmail.com (J.W.); qbtao166@163.com (T.D.); cate_yejt@163.com (J.Y.); 2 College of Life Sciences, Guangxi Normal University, Guilin 541006, China

**Keywords:** *Abies ziyuanensis*, rhizosphere, soil fungi, functional molecular ecological network, FUNGuild database

## Abstract

First discovered in 1977, *Abies ziyuanensis* (Pinaceae) is listed as an endangered species by the International Union for Conservation of Nature (IUCN); its population continues to decline. Analyzing the rhizospheric fungal communities in the two largest populations of *A. ziyuanensis* within the Yinzhu Laoshan National Nature Reserve in Guangxi Zhuang Autonomous Region, China, and factors influencing the rhizosphere will establish a theoretical framework for the reintroduction of *A. ziyuanensis*. This study used ITS rRNA gene high-throughput sequencing and statistical data analysis to compare the functional diversity and structure of the molecular ecological network of rhizospheric fungal communities between *A. ziyuanensis* populations in Yinzhu Laoshan mountain in Guangxi at two sites, Shenbaotang and Sanjiaohutang. A total of 1755 OTUs were identified from the rhizospheric samples of 30 *A. ziyuanensis*; these were classified into seven trophic modes and 81 functional guilds. The most important functional types corresponded to the fungal phyla Ascomycota, Mortierellomycota, and Basidiomycota. Changes in the structure of the functional molecular ecological network of the rhizospheric fungal communities of *A. ziyuanensis* were mainly related to soil nutrient conditions and soil water content, with AK and TK being the most critical. The functional molecular ecological networks of the rhizosphere of *A. ziyuanensis* differed among populations; distinct functional-gene profiles were detected in the rhizosphere microbiomes of different *A. ziyuanensis* populations. These findings provide insight into the role of unique rhizospheric fungi in the growth and environmental adaptation of *A. ziyuanensis*, and for the discovery of superior rhizosphere-promoting fungi.

## 1. Introduction

Rhizosphere is a small ecosystem characterized by a diverse microbial community with intricate interactions, serving as a secondary genomic reservoir for plants [1]. Rhizospheric soil refers to the soil influenced by exudates of plant roots [2]. Fungi are integral components in the material cycling and energy flow within ecosystems [3]. Fungi exhibit diverse species compositions and distribution patterns under varying environmental conditions, resulting in complex fungal communities [4,5]. A functional group of fungi comprises a set of species that utilize environmental resources in a similar way [6]. Soil fungi can be classified into pathogenic, saprotrophic, and symbiotic fungi based on nutritional strategies, each group playing diverse roles in the soil ecosystem [7]. For example, saprotrophic fungi can enhance plant phosphorus use efficiency [8], and they are still the main decomposers in soil, playing an important regulatory role in carbon and nitrogen cycling [9]. Symbiotic fungi serve as a critical component of ecological systems, with mycorrhizal fungi playing a particularly vital role. Approximately 92% of plants on Earth rely on symbiotic partnerships with mycorrhizal fungi to enhance growth and withstand threats such as pathogens and drought conditions [10,11]. At the same time, mycorrhizal fungi improve soil nutrient conditions by mobilizing sources of nutrients obtained from plants into the soil [12]. Pathogenic fungi contribute to the breakdown of living organisms (e.g., animals, plants, and microorganisms), with extreme cases leading to complete plant mortality [13,14]. The coexistence of fungi with distinct nutritional roles enhances the functional diversity of soil communities, which profoundly affects soil ecosystem processes and is important for maintaining ecosystem health [10]. Due to synergistic or antagonistic interactions in resource acquisition and spatial occupancy, soil microorganisms do not occur in isolation but instead constitute a highly interconnected ecological network [15,16]. Phylogenetic-based molecular ecological network analysis offers a powerful tool for studying the interplay among distinct functional groups in a fungal community [17,18]. The phylogenetic relationships between network topology, module members, and nodes (populations) will distinguish whether various taxa are the most important microorganisms in ecological networks [19]. The presence of keystone taxa significantly influences community composition and may even dictate the covariance of functional traits across species interactions [19]. Therefore, identifying the most important taxa of fungal communities and using functional annotation analysis can improve researchers’ understanding of the structural patterns and functional diversity of fungal communities.

*Abies ziyuanensis* (Pinaceae) is a coniferous evergreen tree, which was first discovered in 1977 in the Yinzhu Laoshan Mountains in Guangxi, China, and then successively discovered in Sunhuang Mountain in Xinning County, Hunan Province [20,21]. The two populations with the widest distribution of existing natural populations of *A. ziyuanensis* are Shenbaotang (SBT) and Sanjiaohutang (SJHT) in Yinzhulaoshan Nature Reserve, Ziyuan County, Guilin, Guangxi, China. In the past few decades, populations of wild *A. ziyuanensis* have continued to decline [22]. In 1998, the International Union for Conservation of Nature listed this species as a globally protected species. In 1999, it was listed as a first-level protected plant in the National List of Key Protected Plants in China. Past research on *A. ziyuanensis* has mainly focused on genetic diversity [23], potential habitable zone prediction [24], the analysis of genomic microsatellite sequence characteristics [25], and other aspects of this species. Although these studies predict a close relationship exists between the sharp decline in the number of *A. ziyuanensis* and certain factors, the exact reasons for the decline remain unclear. Plant-derived root metabolites stimulate the growth and activity in their rhizospheric (the portion of soil affected by root exudates) microbial community [26,27]. In contrast, microbial communities in the rhizosphere play a vital role in plant well-being by supplying nutrients, promoting growth hormones, controlling harmful pathogens, and increasing resistance to environmental challenges such as drought, salinity, heat, and frost [28,29]. This indicates that various forms of interactions exist between plants and microorganisms in the rhizosphere, such as reciprocity, symbiosis, and pathogenicity. Xie et al. [30] studied the soil of three high-altitude wetlands located in the northeastern, southeastern, and central Hengduan Mountains of the Qinghai–Tibet Plateau by collecting eight soil samples with a depth of 0–20 cm from each wetland. The functional annotation of fungi by Fungi Functional Guild (FUNGuild) 1.0 software showed that saprotrophic fungi were the most abundant type among the three wetlands. Further analysis of the microbial phylogenetic molecular ecological network indicated that saprotrophic fungi are key taxa in the three fungal networks within wetland systems, while symbiotic fungi and phytotrophic fungi exhibit distinct functional roles in the fungal networks of different wetlands. Therefore, understanding the functional processes that shape fungal communities in the rhizosphere and deciphering the functional composition and interactions between plants and fungal communities in the rhizosphere is crucial for maintaining plant growth and productivity.

In Yinzhu Laoshan Nature Reserve, populations of *A. ziyuanensis* are mainly concentrated at two sites, Shenbaotang (SBT) and Sanjiaohutang (SJHT). In this study, these two *A. ziyuanensis* populations were used as the object of study. Based on high-throughput sequencing data of ITS rRNA genes of fungi in the rhizosphere (0–20 cm soil depth) of various populations, FUNGuild functional prediction and functional molecular ecological network (fMEN) analysis were conducted to explore the functional characteristics of fungal communities and fMEN features in the rhizosphere of *A. ziyuanensis*. This also involved an analysis of the main environmental factors in soil that affect the functional characteristics and functional network features of fungi in the rhizospheric soil in order to reveal the interaction mechanism between *A. ziyuanensis* and fungi in Yinzhu Laoshan, and also provide scientific reference for the field occurrence and population protection of *A. ziyuanensis*.

## 2. Materials and Methods

### 2.1. Overview of the Research Area and Collection of Soil Samples

The research site is located in Yinzhu Laoshan National Nature Reserve in Guilin, Guangxi Zhuang Autonomous Region, China, (26°15′05″–19′15″ N, 110°32′42″–35′06″ E), which experiences a subtropical monsoon climate. The average annual temperature, average temperature in January, and extreme low and high temperatures are 13.1 °C, 2.1 °C, −11.9 °C, and 34 °C, respectively. The average annual rainfall is 2065 mm, with a rainy season from March to August, accounting for 71% of the annual precipitation. The mean annual relative humidity stands at 85%. The main landform type is Zhongshan, with a mainly yellow-brown zonal soil developed from granite. *A. ziyuanensis* at the sampling site occurs at an elevation of 1600–1850 m. The local microclimate of habitat occupied by *A. ziyuanensis* is characterized by experiencing frequent cloud cover, high humidity, a long duration of low temperature, and frequent frost and snow in winter [22].

In late July 2023, during the daytime following rainfall, 15 healthy *A. ziyuanensis* plants were randomly selected from the *A. ziyuanensis* distribution sites of SBT and SJHT in this reserve, individuals exhibiting visible disease symptoms or mechanical damage were excluded, the age of the trees is all over 20 years; collection sites were selected to have similar stand conditions (soil type, slope, and slope orientation) and a relatively homogeneous slope surface. The litter and weeds covered around each target tree were removed; next, approximately 500 g of soil was collected with soil core samplers with an inner diameter of 5.0 cm, while a shovel was used to sample soil at a depth of 0–20 cm, after which samples were placed in sterile, self-sealing bags. Three soil samples were collected around the base of each sample tree in a triangular pattern with samples made at 1 m intervals; these three samples were then combined into a single composite soil sample. A total of 30 samples were taken (15 soil samples per site). Sample information was recorded; an insulated box (with built-in biological ice packs) was then used to store the samples and transport them back to the lab. In the laboratory, fresh mixed soil samples were divided into three parts. A soil sample was sieved through a 2 mm steel sieve and promptly stored at −80 °C for subsequent high-throughput sequencing of soil fungi. A fresh soil sample was sieved through a 2 mm steel sieve and promptly stored at 4 °C for later analysis of ammonium nitrogen, nitrate nitrogen, and microbial biomass carbon and nitrogen content. The third sample was air-dried indoors and then sieved through a 0.25 mm sieve before assessing the soil’s physicochemical properties. All sampling tools (soil auger, spade, etc.) were autoclaved before use and re-sterilized with 70% ethanol between each sampling point to prevent cross-contamination.

### 2.2. Determination of Soil Physicochemical Properties

The soil moisture content (SWC) was measured using the gravimetric method (YH-C30002 electronic balance, Sartorius Scientific Instruments, Beijing, China). The pH value was measured using the potentiometric method, with a water-to-soil ratio of 2.5:1, using an ST2100 pH meter (OHAUS Instruments, Shanghai, China). The soil organic carbon (SOC) content was measured using the potassium dichromate oxidation method with external heating. The total nitrogen (TN) content was measured using sulfuric acid digestion followed by the Kjeldahl nitrogen determination method. The contents of ammonium nitrogen (NH_4_^+^-N) and nitrate nitrogen (NO_3_^−^-N) were measured using the indophenol blue colorimetric method and phenol disulfonic acid colorimetric method, respectively (TU-1901 UV visible spectrophotometer, Beijing Purkinje General Instrument Co., Ltd., Beijing, China). The content of available nitrogen (AN) in soil was determined by alkaline hydrolysis diffusion method. The total phosphorus (TP) content in soil was determined using the sodium hydroxide alkali melting molybdenum antimony colorimetric method (TU-1901 UV visible spectrophotometer, Beijing Purkinje General Instrument Co., Ltd.). The content of available phosphorus (AP) was determined using the sodium bicarbonate/sodium fluoride hydrochloric acid extraction molybdenum antimony colorimetric method (TU-1901 UV visible spectrophotometer, Beijing Purkinje General Instrument Co., Ltd.). The total potassium (TK) content in the soil was measured using NaOH alkali fusion and flame photometry with a TAS-990F flame atomic absorption spectrophotometer produced by Beijing Purkinje General Instrument Co., Ltd. The available potassium (AK) content in soil was measured using ammonium acetate solution extraction flame spectrophotometry with a TAS-990F flame atomic absorption spectrophotometer purchased from Beijing Purkinje General Instrument Co., Ltd. Soil microbial biomass carbon (MBC) and nitrogen (MBN) were extracted using chloroform fumigation and measured by a carbon nitrogen analyzer (Sartorius Scientific Instruments) following the method of Bao [31].

### 2.3. Rhizospheric Soil Fungal DNA Extraction, PCR Amplification, and High-Throughput Sequencing

An EZNA™ Mag-Bind Soil DNA Kit (M5635-02, OMEGA, Cambridge, MA, USA) was used to extract DNA from 30 samples, detect DNA integrity using 2% agarose gel electrophoresis, followed by the use of a Qubit^®^ 4.0 DNA detection kit (Q33238, ThermoFisher, Waltham, MA, USA) to precisely measure genomic DNA and determine the appropriate amount to add to the PCR reaction. Next, PCR amplification (ETC 811, Beijing Dongsheng Innovation Biotechnology Co., Beijing, China) was used to amplify the ITS3-ITS4 gene fragment of *A. ziyuanensis* rhizospheric soil fungal ITS twice, using primers ITS3F (GCATCGATGAAGAACGCAGC) and ITS4R (TCCTCCGCTTATTGATATGC). The two round PCR amplification system was as follows: 2× Hieff^®^ Robust PCR Master Mix (10105ES03, Yeasen, Gaithersburg, MD, USA) 15 µL, Bar-PCR primer F 1 µ, Primer R 1 µ, PCR products 10–20 ng, H_2_O 9–12 µL, total volume 30 µL. The first round of PCR conditions were as follows: 94 °C, 3 min → (94 °C, 30 s → 45 °C, 20 s → 65 °C, 30 s) 5 → (94 °C, 20 s → 55 °C, 20 s → 72 °C, 30 s) 20 → 72 °C, 5 min → 10 °C until stopped. The second round of PCR reaction conditions were as follows: 95 °C, 3 min → (94 °C, 20 s → 55 °C, 20 s → 72 °C, 30 s) 5 → 72 °C, 5 min →10 °C until stopped, reaching the PCR amplification conditions. The library size was detected using 2% agarose gel electrophoresis, and the library concentration was measured using a Qubit 4.0 fluorescence quantitative instrument (Q33238, ThermoFisher), all samples were mixed in equal 1:1 amounts. Entrust Biotechnology Co., Ltd. (Shanghai, China) provided an Illumina MiseqPE300 platform for library construction and high-throughput sequencing.

### 2.4. Bioinformatics Analysis

The obtained raw image data file was transformed into raw sequences (Sequenced Reads) through base calling analysis using an Illumina MiseqTM/HiseqTM (Sangon Biotechnology Co., Ltd., Shanghai, China). Cutadapt 1.18 software was used to remove primer adapter sequences; then, the PEAR 0.9.8 software was utilized to merge paired reads into a sequence, leveraging the overlap between paired-end reads. Then, samples were identified and distinguished according to barcode label sequences to obtain each bit of sample data. Finally, PRINSEQ 0.20.4 software was utilized to conduct quality control filtering on each sample’s data, yielding valid data for each sample. Next, Usearch 11.0.667 software was used to cluster non-repetitive sequences (excluding single sequences) into operational taxonomic units (OTUs) at 97% similarity. Chimeras were removed during the clustering process, representative sequences of the OTUs were obtained, and mothur 1.43.0 (classify.seq) software was used to combine representative OTU sequences with Silva (https://www.arb-silva.de/) (accessed on 21 April 2024). Species annotation was performed through database comparison. After merging the OTU table and the species annotation information table, the trophic mode and guild of each sample fungal community were compared using the FUNGuild 1.0 database.

Constructing a fungal fMEN based on OTU data can be completed by detecting rhizospheric soil fungal community trophic and functional guilds [18]. First, a logarithmic (log10) transformation was applied to the OTU data using the MENA platform, enabling the calculation of the Pearson correlation between any two OTUs. A fungal fMEN was then constructed using random matrix theory. The network topology parameters were calculated, including network nodes, edges, average degree of connectivity, average clustering coefficient, average path distance, and modularity. Based on intra-module connectivity (*Zi*) and inter-module connectivity (*Pi*), network nodes can be classified into four categories: (1) module hubs (*Zi* ≥ 2.5 and *Pi* < 0.62); (2) connectors (*Zi* < 2.5 and *Pi* ≥ 0.62); (3) network hubs (*Zi* ≥ 2.5 and *Pi* ≥ 0.62); and (4) peripheral nodes (*Zi* < 2.5 and *Pi* < 0.62). Among them, module hubs, connectors, and network hubs are important functions in the fMEN. The ecological network structure of functional molecules was then visualized using Gephi-0.9.2 software.

### 2.5. Statistical Analysis of Data

A *t*-test was utilized to detect the significant differences in soil physicochemical properties between the two plots, while a non-parametric Wilcox test was conducted to test the significant differences in rhizospheric soil fungal trophic modes and functional guilds between the two plots. Pearson correlation analysis was employed to analyze the effects of parameters related to the soil physicochemical properties for soil at *A. ziyuanensis* sites on the trophic modes, dominant functional groups, and differential functional groups of fungal communities. The hierarchical clustering method was utilized to cluster representative modules of fungal fMEN, while Pearson correlation analysis was performed to analyze the correlation between parameters of soil physicochemical properties and representative modules. Relevant calculations and graphs were completed in Excel 2019 and GraphPad Prism 8.3 software.

## 3. Results

### 3.1. Soil Physicochemical Properties of the Rhizosphere of A. ziyuanensis

A *t*-test (Table 1) found no significant differences in SWC, pH, TN, AN, NO_3_^−^-N, NH_4_^+^-N, SOC, TP, AP, MBC, and MBN between the soils of the two populations of *A. ziyuanensis* (*p* > 0.05). However, TK and AK were significantly higher at the SBT site than at the SJHT (*p* < 0.05).

### 3.2. Trophic Modes and Functional Guilds of Rhizospheric Soil Fungal Communities of A. ziyuanensis

Based on the FUNGuild database, which categorizes fungi based on their ecological function, the fungal communities in all rhizospheric soil samples associated with *A. ziyuanensis* were classified into various trophic modes and functional guilds. Among the 1755 detected OTUs (accounting for 60.14% of the total OTUs), the OTUs were categorized into seven trophic modes and 81 functional guilds. The seven fungal trophic modes are: Symbiotroph, Symbiotroph–Saprotroph, Saprotroph, Pathotroph–Symbiotroph, Pathotroph–Saprotroph–Symbiotroph, Pathotroph–Saprotroph, and Pathotroph (Table 2). Notably, functioning as a Symbiotroph is the dominant trophic mode for fungi in the rhizosphere of *A. ziyuanensis* (20.97–22.00%).

**Figure 1:**
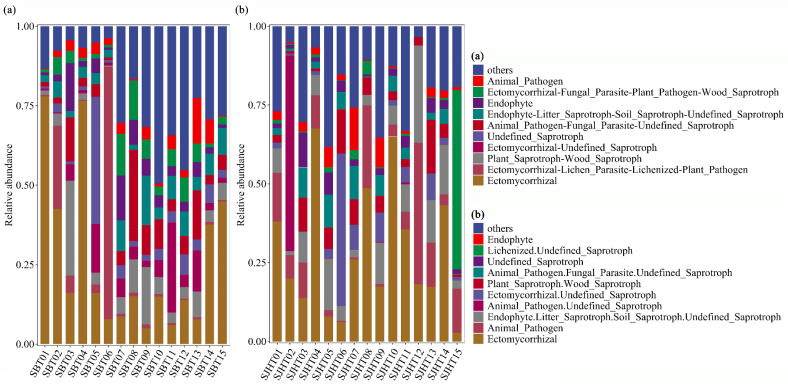
Relative abundance of functional guilds in the rhizosphere fungal communities of the (**a**) Shenbaotang and (**b**) Sanjiaohutang sites. Note: SBT01 to SBT15 and SJHT01 to SJHT15 represent samples from the rhizosphere of 15 plants analyzed at the two sites, the Shenbaotang (SBT) and Sanjiaohutang sites (SJHT).

Among the 81 fungal functional guilds, the top 10 guilds with relatively high abundance were considered as the dominant functional guilds (Figure 1). The Animal_Pathogen, Endophyte, Endophyte-Litter_Saprotroph-Soil_Saprotroph-Undefined_Saprotroph, Animal_Pathogen-Fungal_Parasite-Undefined_Saprotroph, Undefined_Saprotroph, Ectomycorrhizal-Undefined_Saprotroph, Plant_Saprotroph-Wood_Saprotroph, and Ectomycorrhizal guilds were the common dominant functional guilds in both SBT and SJHT sites. Unique dominant functional guilds in the SBT site included Ectomycorrhizal-Fungal_Parasite-Plant_Pathogen-Wood_Saprotroph and Ectomycorrhizal-Lichen_Parasite-Lichenized-Plant_Pathogen (Figure 1a). Unique dominant functional guilds in the SJHT site included Lichenized.Undefined_Saprotroph and Animal_Pathogen.Undefined_Saprotroph (Figure 1b).

### 3.3. Differences in Trophic Modes and Functional Guilds of Rhizospheric Soil Fungal Communities of A. ziyuanensis

**Figure 2:**
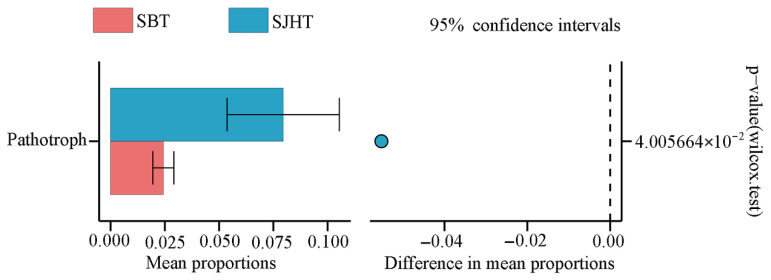
Differential analysis of trophic modes in the rhizospheric soil fungal communities. Note: Site names were the Shenbaotang (SBT) and Sanjiaohutang sites (SJHT).

A Wilcoxon test indicated that the relative abundance of the Pathotroph trophic mode was significantly higher at the SJHT site compared to the SBT (*p* < 0.05; Figure 2). Conversely, several functional guilds, including Bryophyte Parasite-Dung Saprotroph-Ectomycorrhizal-Fungal Parasite-Leaf Saprotroph-Plant Parasite-Undefined Saprotroph-Wood Saprotroph, Dung Saprotroph-Plant Saprotroph-Wood Saprotroph, Ectomycorrhizal-Lichen Parasite-Lichenized-Plant Pathogen, Endomycorrhizal-Plant Pathogen-Undefined Saprotroph, NULL, and Plant Parasite-Wood Saprotroph, were significantly more frequent at the SBT site than in the SJHT (*p* < 0.05). Additionally, the fungal functional guilds of SJHT exhibited significantly higher relative abundances of Animal Pathogen, Arbuscular Mycorrhizal, Dung Saprotroph, Ectomycorrhizal-Undefined Saprotroph-Wood Saprotroph, and Plant Saprotroph than those of SBT (*p* < 0.05; Table 3).

### 3.4. The Influence of Soil Physicochemical Properties on the Trophic Modes and Functional Groups of Fungal Communities in the Rhizosphere of A. ziyuanensis

**Figure 3:**
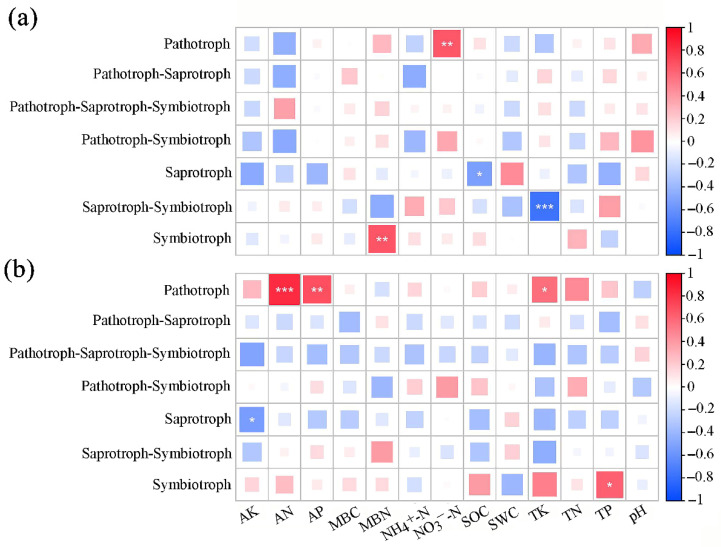
Pearson correlation analysis of relative abundance of functional trophic genes of rhizospheric soil fungi and soil physicochemical factors for sites (**a**) Shenbaotang and (**b**) Sanjiaohutang. Note: Red and blue represent positive and negative correlations between two variables, respectively. The darker the color, the closer the relationship, and *, **, and *** represent *p* < 0.05, *p* < 0.01, and *p* < 0.001, same below; AK, available potassium; AN, available nitrogen; AP, available phosphorus; MBC, microbial biomass carbon; MBN, microbial biomass nitrogen; NH_4_^+^-N, ammonium nitrogen; NO_3_^−^-N, nitrate nitrogen; SOC, soil organic carbon; SWC, soil water content; TK, total potassium; TN, total nitrogen; TP, total phosphorus. * *p* < 0.05; ** *p* < 0.01; *** *p* < 0.001.

Pearson’s correlation analysis showed that the Symbiotroph fungal trophic mode of the SBT site was highly significant and positively correlated with MBN (*p* < 0.01), Pathotroph was highly significant and positively correlated with NO_3_^−^-N (*p* < 0.01), and Saprotroph was significant and positively correlated with SOC (*p* < 0.05). Saprotroph–Symbiotroph showed an extremely significant negative correlation with TK (*p* < 0.001), and the other trophic types were not significantly correlated with any soil physicochemical factors (*p* > 0.05; Figure 3a). The Pathotroph trophic mode of SJHT showed an extremely significant positive correlation with AN (*p* < 0.001), a highly significant positive correlation with AP (*p* < 0.05), significant positive correlation (*p* < 0.05) with TK, a significant negative correlation (*p* < 0.05) between Saprotroph and AK, and a significant positive correlation (*p* < 0.05) between Symbiotroph and TP, while the remaining trophic types had no significant correlation (*p* > 0.05) with any soil physicochemical factors (Figure 3b).

**Figure 4:**
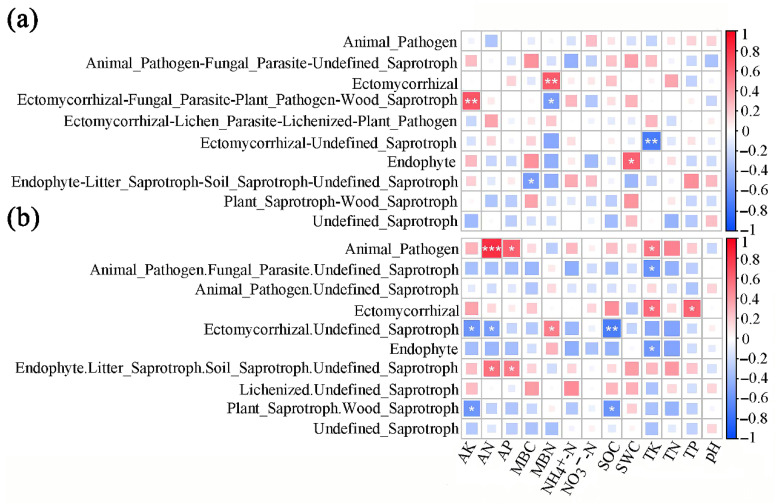
Pearson correlation analysis of relative abundance of dominant functional group genes of rhizospheric soil fungi and soil physicochemical factors for sites (**a**) Shenbaotang and (**b**) Sanjiaohutang. Note: AK, available potassium; AN, available nitrogen; AP, available phosphorus; MBC, microbial biomass carbon; MBN, microbial biomass nitrogen; NH_4_^+^-N, ammonium nitrogen; NO_3_^−^-N, nitrate nitrogen; SOC, soil organic carbon; SWC, soil water content; TK, total potassium; TN, total nitrogen; TP, total phosphorus. * *p* < 0.05; ** *p* < 0.01; *** *p* < 0.001.

The Ectomycorrhizal functional guild of the SBT site showed a highly significant positive correlation with MBN (*p* < 0.01), while Ectomycorrhizal-Fungal_Parasite-Plant_Pathogen-Wood_Saprotroph showed a highly significant positive correlation with AK (*p* < 0.01) and a highly significant negative correlation with MBN (*p* < 0.05). Moreover, the Ectomycorrhizal-Undefined_Saprotroph guild was highly significantly negatively correlated with TK (*p* < 0.01), the Endophyte guild was significantly positively correlated with SWC (*p* < 0.05), and the Endophyte-Litter_Saprotroph-Soil_ Saprotroph-Undefined_Saprotroph was significantly negatively correlated with MBC (*p* < 0.05) (Figure 4a). In addition, the Animal_Pathogen functional guild of the SJHT site was extremely significantly positively correlated with AN (*p* < 0.001), and significantly positively correlated with AP and TK (*p* < 0.05). Furthermore, the Animal_Pathogen.Fungal_Parasite.Undefined_Saprotroph functional guild showed significant negative correlations with TK (*p* < 0.05). The Ectomycorrhizal guild showed a significant positive correlation with TK and TP (*p* < 0.05), while the Ectomycorrhizal.Undefined-Saprotroph guild was significantly negatively correlated with AK and AN (*p* < 0.05), significantly positively correlated with MBN (*p* < 0.05), and highly significantly negatively correlated with SOC (*p* < 0.01). The Endophyte guild was significantly negatively correlated with TK (*p* < 0.05). In addition, the Endophyte.Litter_Saprotroph.Soil_Saprotroph.Undefined_Saprotroph guild were significantly and positively correlated with AN and AP (*p* < 0.05). In addition, the Plant_Saprotroph.Wood_Saprotroph guild was significantly and negatively correlated with AK and SOC (*p* < 0.05) (Figure 4b).

**Figure 5:**
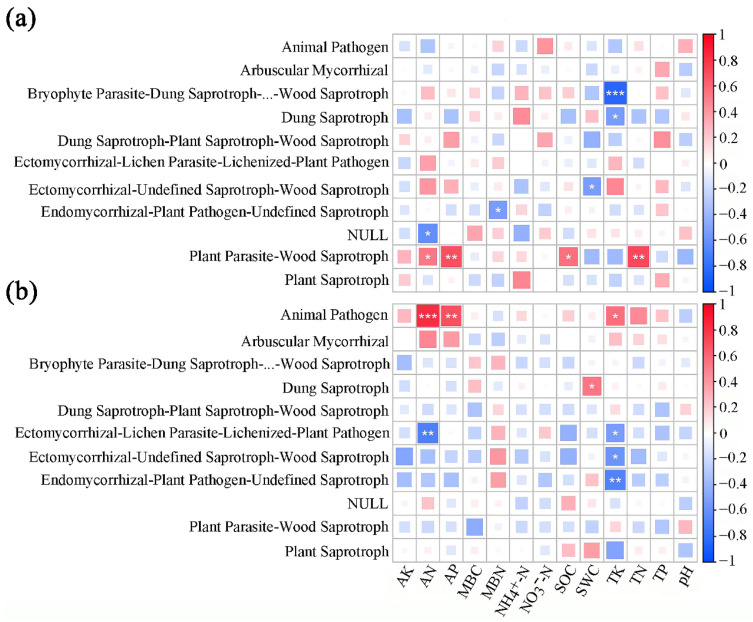
Pearson correlation analysis of relative abundance of differential functional group genes of rhizospheric soil fungi and soil physicochemical factors for sites (**a**) Shenbaotang and (**b**) Sanjiaohutang. Note: AK, available potassium; AN, available nitrogen; AP, available phosphorus; MBC, microbial biomass carbon; MBN, microbial biomass nitrogen; NH_4_^+^-N, ammonium nitrogen; NO_3_^−^-N, nitrate nitrogen; SOC, soil organic carbon; SWC, soil water content; TK, total potassium; TN, total nitrogen; TP, total phosphorus. Bryophyte Parasite-Dung Saprotroph-…-Wood Saprotroph: Bryophyte Parasite-Dung Saprotroph-Ectomycorrhizal-Fungal Parasite-Leaf Saprotroph-Plant Parasite-Undefined Saprotroph-Wood Saprotroph. * *p* < 0.05; ** *p* < 0.01; *** *p* < 0.001.

The Bryophyte Parasite-Dung Saprotroph-Ectomycorrhizal-Fungal Parasite-Leaf Saprotroph-Plant Parasite-Undefined Saprotroph-Wood Saprotroph functional guild of saprophytes at the SBT site was extremely significantly negatively correlated with TK (*p* < 0.001), while the Dung Saprotroph guild was significantly negatively correlated with TK (*p* < 0.05), and the Ectomycorrhizal-Undefined Saprotroph-Wood Saprotroph guild was significantly negatively correlated with SWC (*p* < 0.05). Furthermore, the Endomycorrhizal-Plant Pathogen-Undefined Saprotroph was significantly negatively correlated with MBN (*p* < 0.05), the NULL guild was significantly negatively correlated with AN (*p* < 0.05), and the Plant Parasite-Wood Saprotroph guild was significantly positively correlated with AP and TN (*p* < 0.01) and significantly positively correlated with AN and SOC (*p* < 0.05) (Figure 5a). The Animal Pathogen guild of the SJHT site was extremely significantly positively correlated with AN (*p* < 0.001), highly significant positively correlated with AP (*p* < 0.01), and significantly positively correlated with TK (*p* < 0.05). The Dung Saprotroph guild was significantly positively correlated with SWC (*p* < 0.05), while the Ectomycorrhizal-Lichen Parasite-Lichenized-Plant Pathogen guild was highly significantly negatively correlated with AN (*p* < 0.01) and significantly negatively correlated with TK (*p* < 0.05). In addition, the Ectomycorrhizal-Undefined Saprotroph-Wood Saprotroph guild was significantly negatively correlated with TK (*p* < 0.05), while the Endomycorrhizal-Plant Pathogen-Undefined Saprotroph guild was highly significantly negatively correlated with TK (*p* < 0.01; Figure 5b).

**Figure 6:**
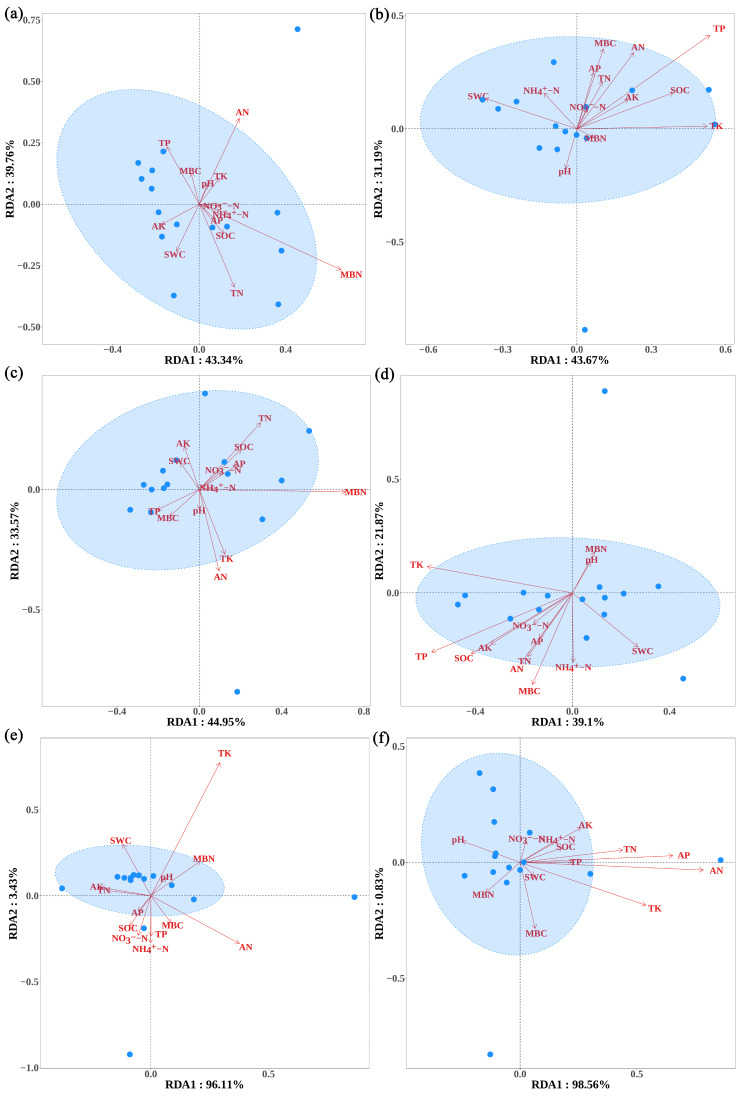
Redundancy analysis (RDA) based on rhizospheric soil fungal trophic modes for the (**a**) Shenbaotang and (**b**) Sanjiaohutang sites; RDA based on the dominant functional groups of rhizospheric soil fungi for (**c**) Shenbaotang and (**d**) Sanjiaohutang; RDA based on differential functional groups of rhizospheric soil fungi for (**e**) Shenbaotang and (**f**) Sanjiaohutang. Note: blue dots indicate the sample positions of the respective communities.

Redundancy analysis (RDA) showed that MBN, AN, and TN had the greatest impact on the trophic modes of the SBT fungal community (Figure 6a), while TP and TK had the greatest impact on the trophic modes of the SJHT fungal community (Figure 6b). MBN, AN, TN, and TK exerted the strongest impact on the dominant functions of the SBT fungal community (Figure 6c), while the fungal community of SJHT was the most strongly influenced by TP, TK, SOC, and MBC (Figure 6d). TK and AN had the greatest effect on the differential functions of the SBT fungal community (Figure 6e), while the dominant factors affecting the SJHT fungal community were AN, AP, and TK (Figure 6f).

### 3.5. Characteristics of Fungal fMEN Structure in the Rhizosphere of A. ziyuanensis

The analysis of network topology parameters (Table 4) shows that the threshold of the ecological network of two rhizospheric soil fungal functional molecules was 0.690, and the R2 is 0.7, which conforms to the power law and can be analyzed in more detail.

**Figure 7:**
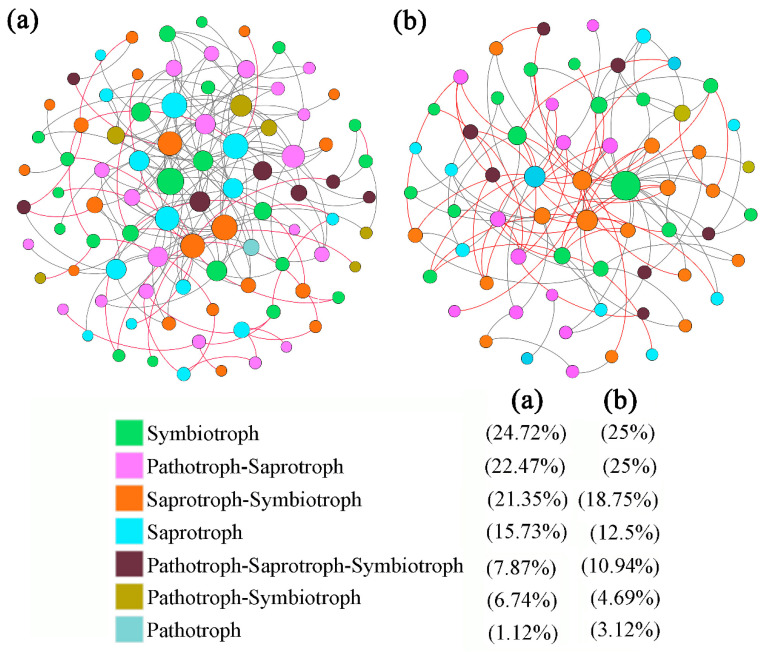
Rhizospheric soil fungal functional molecular ecological networks for the sites (**a**) Shenbaotang and (**b**) Sanjiaohutang. Note: The color of nodes indicates the functional trophic type of fungi; red and gray lines represent positive and negative interactions between nodes, respectively.

The comparative analysis of topological parameters of rhizospheric soil fungal fMENs at the two sites analyzed in this study (Table 4, Figure 7a,b) showed that SBT site exhibited a greater number of total nodes, more total links, a higher number of negative connections, a greater average degree of connection, and a longer average path distance compared with the SJHT site. The average clustering coefficients were all greater at the SJHT site than at the SBT site. The modularity index was equal at both sites. In addition, the positive connections of the rhizospheric soil fungal fMENs of the two accounted for 21.74% and 52.17% of their total edges, respectively. Moreover, the negative connections accounted for 78.26% and 47.83% of their total edges, respectively. In addition, the Symbiotroph (24.72%, 25%), Pathotroph-Symbiotroph (22.47%, 25%), and Saprotroph–Symbiotroph (21.35%, 18.75%) functional guilds represented the largest share of total nodes in the rhizospheric soil fungal fMENs of the two sites.

### 3.6. Topological Roles of fMEN Nodes of Fungi in the Rhizosphere of A. ziyuanensis

**Figure 8:**
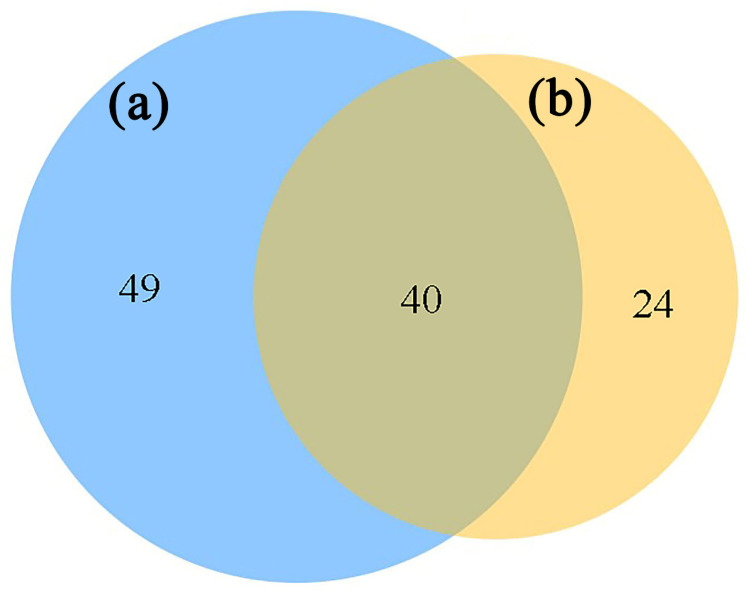
Venn diagram of node distribution in the rhizosphere fungal functional molecular ecological networks for sites (**a**) Shenbaotang and (**b**) Sanjiaohutang.

**Figure 9:**
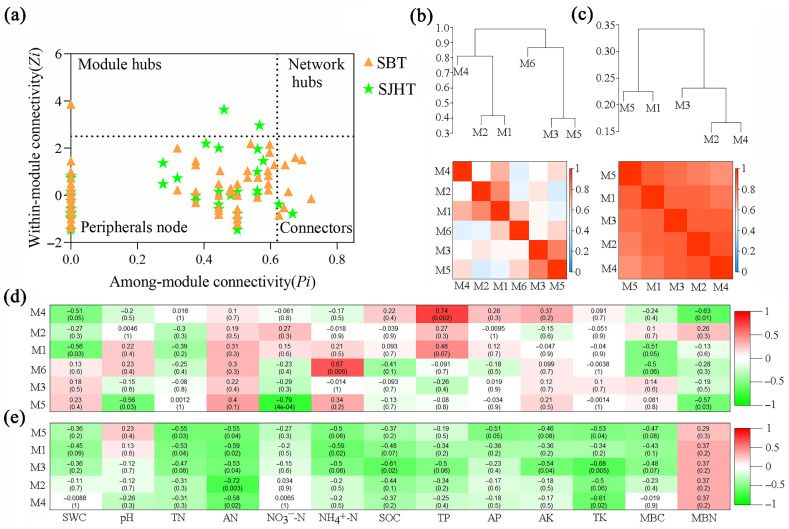
(**a**) Topological role distribution map of rhizospheric soil fungal functional molecular ecological network (fMEN) nodes sites for Shenbaotang (SBT) and Sanjiaohutang (SJHT), (**b**,**c**) hierarchical structure of fMEN modules, and (**d**,**e**) their correlation with soil physicochemical properties heatmap. Note: Red and blue in (**b**,**c**) indicate high low correlations, respectively; red and green in (**d**,**e**) indicate highly positive and highly negative correlations, respectively. Numbers without and with parentheses in each figure represent the correlation coefficient (*r*) and significance (*p*), respectively. Mn: Module *n* for modules 1 to 6.

The statistical analysis of nodes (at the OTU level) in the fMEN of fungi in the rhizosphere of *A. ziyuanensis* shows that a total of 113 non-duplicated nodes existed in the distribution areas of the two *A. ziyuanensis* populations (Figure 8) analyzed here. Among them, there are 40 shared nodes, and the sites SBT and SJHT have 49 and 24 unique nodes, respectively. Further statistical analysis (Figure 9a) revealed that the peripheral nodes of both fungal fMENs were dominant (SBT: 91.01%; SJHT: 93.75%), while there were no network hubs. The SBT site has eight key nodes, namely one module hub and seven connectors, belonging to six trophic types (Table 5): Symbiotroph (2 key nodes), Pathotroph–Saprotroph–Symbiotroph (1), Saprotroph–Symbiotroph (1), Saprotroph (2), Pathotroph–Saprotroph (1), Pathotroph (1) (Table 5). Furthermore, the SJHT site has four key nodes, including two module hubs and five connectors, belonging to two trophic types (Table 5): Symbiotroph (1) and Saprotroph–Symbiotroph (3).

In addition, among the eight key nodes in the ecological network of the SBT site, six belong to Ascomycota, one belongs to Basidiomycota, and one belongs to Mortierellomycota. Compared with the nodes at the SJHT site, the SBT site has one unique key phylum, Mortierellomycota; three of the four key nodes at the SJHT belong to Ascomycota and one belongs to Basidiomycota (Table 5).

### 3.7. The Influence of Soil Physicochemical Properties on the Ecological Network Structure of Fungal Functional Molecules

From the viewpoint of the module hierarchy among the fMENs of rhizospheric soil fungi, at the SBT site closely linked modules included modules 1 and 2 as well as 3 and 5, along with module 4, which was closely linked to modules 1 and 2. Further, module 6 was relatively closely linked to both modules 3 and 5, while modules 4 and 6 were significantly differentiated (Figure 9b). For the SJHT site, closely linked modules included 1 and 5 as well as 2 and 4. Moreover, module 3 was relatively closely linked to both modules 2 and 4, while it was significantly differentiated from modules 1 and 5 (Figure 9c).

The impact of soil physicochemical properties on the ecological network structure of functional molecules of each rhizospheric soil fungal community varied (Figure 9). At the SBT site (Figure 9d), SWC was significantly negatively correlated with module 1 (M1, *r* = −0.56, *p* = 0.03); TP and MBN were significantly positively and significantly negatively correlated with module 4, respectively (M4, *r* = 0.74, *p* = 0.002; *r* = −0.63, *p* = 0.01, respectively); pH, NO_3_^−^-N, and MBN were significantly negatively correlated with module 5 (M5, *r* = −0.56, *p* = 0.03; *r* = −0.79, *p* = 4e-04; *r* = −0.57, *p* = 0.03, respectively); and NH_4_^+^-N was significantly positively correlated with module 6 (M6, *r* = 0.67, *p* = 0.006). At the SJHT site (Figure 9e), TN, AN, and NH_4_^+^-N were significantly negatively correlated with M1 (r = −0.53, *p* = 0.04, *r* = −0.59, *p* = 0.02, and *r* = −0.59, *p* = 0.02, respectively); AN was significantly negatively correlated module 2 (*r* = −0.72, *p* = 0.003); AN, SOC, AK, and TK were significantly negatively correlated with module 3 (*r* = −0.53, *p* = 0.04, *r* = −0.61, *p* = 0.02, *r* = −0.54, *p* = 0.04, and *r* = −0.68, *p* = 0.005, respectively); AN and TK were significantly negatively correlated with M4 (*r* = −0.58, *p* = 0.02, *r* = −0.61, *p* = 0.02); TN, AN, and TK were significantly negatively correlated with M5 (*r* = −0.55, *p* = 0.03, *r* = −0.55, *p* = 0.04, *r* = −0.53, *p* = 0.04, respectively). Thus, soil SWC, pH, NO_3_^−^-N, NH_4_^+^-N, TP, and MBN were the dominant factors affecting the ecological network of functional molecules of rhizospheric soil fungi at the SBT site. Additionally, TN, AN, NH_4_^+^-N, SOC, AK, and TK were the dominant factors affecting the ecological network of functional molecules of rhizospheric soil fungi at the SJHT site.

## 4. Discussion

### 4.1. Analysis of Functional Groups of Fungal Communities in the Rhizosphere of A. ziyuanensis

The fungal community in soil plays an important role in providing nutrients to plants, promoting soil nutrient cycling, and enhancing ecosystem functions [32]. Fungi adapt to changes in their living environment by adopting various nutritional methods, which is a unique advanced survival strategy of fungi [33]. Saprotrophic fungi mainly obtain nutrients by decomposing dead plants or animals, pathological fungi acquire nutrients through damaging host cells, and symbiotic fungi mainly obtain nutrients by exchanging resources with host cells [34]. The current study revealed that the dominant trophic modes of fungi in the rhizosphere of plants of *A. ziyuanensis* at various sites was symbiotic trophic (Table 2). However, the research by Yang et al. [35] showed that a saprotrophic mode is the dominant trophic mode of soil fungi, which is inconsistent with the present study. However, research by Xi et al. [36] found that in the plantation forests of native tree species in the South Asian tropical region, the saprotrophic trophic mode is dominant for soil fungi, while the symbiotic trophic mode is dominant for soil fungi in *Eucalyptus urophylla* forest. Wen et al. [37] found through studying the characteristics of changes in soil conditions and differences in fungal community structure and function among different forest types in subtropical regions, that *Cunninghamia lanceolata* forests are mainly saprotrophic, while *Pinus massoniana* forests, *Castanopsis eyrei* forests, and mixed forests are mainly symbiotic. Pearson correlation analysis revealed that symbiotic fungi were strongly positively correlated with MBN and TP (*p* < 0.05) (Figure 3a,b). Therefore, MBN and TP in soil may be the most significant factors influencing the symbiotic trophic types of the rhizosphere of *A. ziyuanensis*. Plant communities growing under poor nutritional conditions rely more on mycorrhizal fungi mediation than other plant communities because the fungi absorb and assimilate soil nutrients, making them available to plant communities [38]. This may be among the reasons for the relatively high abundance of symbiotic nutritional fungi in areas with *A. ziyuanensis*. When the proportion of saprotrophic and parasitic fungi is higher than that of symbiotic fungi, the stress resistance of trees will continue to weaken, leading to the occurrence of diseases [39]. Rich communities of symbiotic fungi that provide nutrients to plants can effectively improve the absorption efficiency of nitrogen and phosphorus by plants, combat plant pathogens through antagonistic effects, reduce the production of pathological fungi, inhibit the colonization of pathogens, and thus reduce the degree of plant diseases [40]. Therefore, increasing the proportion of symbiotic fungi that provide nutrients to plants can help enhance the stress resistance of *A. ziyuanensis*. The fact that the proportion of saprotrophic and pathogenic bacteria is higher than that of symbiotic bacteria is related to the occurrence of plant diseases [33]. In the present study, a Wilcox test found that the relative abundance of Pathotroph in the SJHT fungal community was significantly higher than that in SBT (*p* < 0.05; Figure 2), which may be a survival strategy of fungi in relatively poor nutrient environments.

In addition, as fungi that symbiotically coexist with plant roots, ectomycorrhizal fungi play a crucial role in enhancing plant stress resistance, regulating the microenvironment of the rhizosphere, and stabilizing plant communities [41]. When the abundance of ectomycorrhizal fungi reaches a certain level, they can promote the secondary succession process of forests by altering the composition of the plant community that coexists with plant roots [42]. In the present study, ectomycorrhizal fungi were not only the primary functional guild in the rhizosphere of the SBT site but also in the rhizosphere of the SJHT site. Chen et al. [43] found that the proportion of arbuscular mycorrhizal fungi in the soil of *Eucalyptus urophylla* forests in the tropical region of South Asia was the highest. This finding is consistent with the results of the present study. Ectomycorrhizal fungi can enhance plants’ absorption of nutrients and water [44]. Although relatively high levels and abundance of ectomycorrhizal fungi in soil can increase plant absorption of soil moisture and other nutrients, it can also lead to a decrease in soil fertility. The correlation analysis revealed that the abundance of the Ectomycorrhizal functional guild of the SBT site was significantly positively correlated with MBN (*p* < 0.01; Figure 4a), and the abundance of the Ectomycorrhizal guild of the SJHT site was significantly positively correlated with TK and TP (*p* < 0.05) (Figure 4b).

No significant correlations were detected between NULL and Undefined Saprotroph and soil physicochemical properties, which implies that soil physicochemical properties may have a limited impact on NULL and Undefined Saprotroph. Although NULL and Undefined Saprotroph do not strongly influence the structure of fungal functional communities, they may play subtle roles in fungal dynamics that have not been identified in this study. Future research could explore the functional roles of NULL and Undefined Saprotroph and their potential interactions with *A. ziyuanensis* to better elucidate the survival dynamics of this endangered species.

### 4.2. Functional Molecular Ecological Network Analysis of Fungal Communities in the Rhizosphere of A. ziyuanensis

Functional molecular ecological networks can effectively reflect the intricacy and stability of microbial ecological networks, helping researchers to analyze their key and important functional genes, and have significant implications for research into biodiversity, global change biology, systems microbiology, and ecology [45]. The number of network nodes and connections can characterize the size of a network [19]. The present study found that the total numbers of nodes and connections in the fMEN of the rhizosphere of the *A. ziyuanensis* community at the SBT site were higher than those at the SJHT site (Table 4 and Figure 7), indicating that the scale and complexity of the fMEN of the rhizosphere community of *A. ziyuanensis* at the SBT site were larger and more complex. In addition, the negative connections in the fMEN of the rhizosphere community of *A. ziyuanensis* at the SBT site were higher (Table 4 and Figure 7), indicating stronger competition among fungi in the rhizosphere of *A. ziyuanensis* at the SBT site than at the SJHT site. This may occur because the available and total potassium contents in the rhizosphere of *A. ziyuanensis* at the SJHT site were significantly lower than those at the SHB site (Table 1).

The larger the average connectivity value, the more complex the network becomes, and the higher the average clustering coefficient, the tighter the inter-cluster connections and the higher the organizational orderliness [45]. The present study found that the average connectivity of the SBT site was higher than that of the SJHT, but the average clustering coefficient was lower than that of the SJHT site, indicating that the fMEN of the rhizosphere fungal community of *A. ziyuanensis* at the SBT site was more complex but the orderliness of the community organization was lower (Table 4 and Figure 7). The smaller the average path distance value, the tighter the species are clustered, and the greater the efficiency of transmitting matter, energy, and information; nevertheless, the reaction to interference is also more sensitive [45,46]. The higher the modularity index value, the stronger the ability of the system to resist external interference [45]. In the present study, the average path distance of the fMEN of the rhizosphere fungal community of *A. ziyuanensis* at the SBT site was greater than that at the SJHT site, but the modularity index was lower than that at the SJHT site (Table 4 and Figure 7). This suggests that the response speed of fungi in the rhizosphere of *A. ziyuanensis* at the SBT site was slow and susceptible to external environmental interference, resulting in poor stability of the community structure. Therefore, more important fungi are needed to maintain the stability of the structure of the molecular ecological network. The fungal communities in the rhizosphere *A. ziyuanensis* at the SJHT site were more sensitive to the external environment and responded promptly. When the external environment changes, the community structure can remain relatively stable.

Module hubs and connectors are important species in community structure, playing a crucial role in maintaining community stability. Their removal can cause significant alterations in the functional dynamics of the fungal community [45,46]. The present study found that among the eight key nodes in SBT, six were Ascomycota, one was Basidiomycota, and one was Mortierellomycota. Three of the four key nodes in SJHT were Ascomycota and one was Basidiomycota (Table 5 and Figure 9a). Ascomycota and Basidiomycota play crucial roles in soil nutrient cycling and the function and stability of microbial communities; they are capable of degrading recalcitrant substances such as lignin and keratin. In addition, most fungi in the phylum Ascomycota live saprotrophic lives and are usually abundant in acidic soils. Fungi in the phylum Basidiomycota are mainly saprotrophic or symbiotic trophic and can form mycorrhizae with plants through symbiosis [32]. Eo et al. [47] studied the endophytic fungal communities of four alpine coniferous trees (*Abies nephrolepis*, *Pinus pumila*, *Taxus cuspidata* var. *nana*, and *Thuja koraiensis*) and found that, regardless of the host plant and habitat, taxa in the phylum Ascomycota dominate as endophytic fungi in most host plants and may have certain ecological functions. In the present study, most of the important species at the SBT site and the SJHT site are ascomycetes, which profoundly affect the stability of the fMEN structure. There are similarities and differences in the nutritional patterns of fungal fMENs in the rhizosphere of *A. ziyuanensis* at the two sites analyzed here, and there are also some functional differences between key species. Overall, saprotrophic nutrition is an important function of the fungal community in the rhizosphere of *A. ziyuanensis* (Table 5). Related studies have shown that nitrogen (nitrate, ammonium, and total nitrogen), pH, and water content are important environmental factors that affect fungal communities [30]. In the present study, soil SWC, pH, NO_3_^−^-N, NH_4_^+^-N, TP, and MBN were the dominant factors affecting the fMEN of rhizospheric soil fungi of the SBT site (Figure 9d); TN, AN, NH_4_^+^-N, SOC, AK, and TK were the dominant factors affecting the fMEN of rhizospheric soil fungi at the SJHT site (Figure 9e). These findings indicate that soil nutrient conditions are limiting factors for the fMEN of fungal communities in the rhizosphere of *A. ziyuanensis* in Yinzhu Laoshan.

### 4.3. Research Prospects of Functional Groups of Fungal Communities in the Rhizosphere of A. ziyuanensis

Previous studies have indicated that changes in the rhizosphere fungal community may also be influenced by factors such as altitudinal gradients [48] and temporal dynamics [49,50]. For example, research by Li [48] suggests that altitude significantly affects the stability of soil microbial communities in alpine meadows, with the looseness and instability of fungal molecular ecological network structures generally increasing and then decreasing with rising altitude. However, there was no significant difference in the altitudinal gradient of the Yinzhu Laoshan *A. ziyuanensis* population in the present study (Table S1); as a result, no comparative analysis of different altitudinal gradients was conducted. The impact of altitudinal gradients on different *A. ziyuanensis* populations will be a focus of future research. In addition, Huang et al. [49] investigated variations in nutrient content and microbial quantity in rhizosphere and non-rhizosphere soils of *Camellia oleifera* across different seasons, demonstrating that the trends in nutrient content and microbial quantity in rhizosphere and non-rhizosphere soils were consistent with seasonal changes, displaying an initial increase followed by a decrease from spring to winter. The general trend was summer > autumn > spring > winter, with some local fluctuations. Notably, the nutrient content and microbial quantity in the rhizosphere of *C. oleifera* exhibited a clear rhizosphere enrichment effect in summer and autumn. A report by Yu et al. [50] indicates that the rhizosphere fungal community is also significantly affected by interannual dynamics, indicating that the temporal dynamic changes in microbial activity play a crucial role in plant growth, soil nutrient cycling, and plants’ ability to acquire nutrients. Therefore, whether the change characteristics of the two *A. ziyuanensis* populations during winter are consistent with those during summer, and whether the bulk soil fungal community change characteristics are consistent with those of the rhizospheric soil, still require further investigation.

Some studies have also found that root biomass [51] and associated tree species [52] can affect the rhizosphere community of plants. Our research team has investigated the ecological niche and interspecific associations of dominant tree species in the *A. ziyuanensis* community in Guangxi, revealing that the interspecific associations among dominant tree species in the *A. ziyuanensis* community are weak, featuring high independence and significant niche overlap. This is suggestive of potential intense competition and a relatively unstable community succession stage that is susceptible to external disturbances. Our team is also researching the impact of canopy cover, light, litterfall, and the physiological status, growth rates, and stress markers of *A. ziyuanensis* on its rhizosphere community.

In the present study, the results of RDA further confirm that soil physicochemical properties have a significant impact on the trophic modes, dominant functions, and differential functions of the rhizosphere fungal communities of the two *A. ziyuanensis* populations (Figure 6). However, due to the limitations of microbial sequencing methods, the study’s prediction of the functional aspects of the *A. ziyuanensis* rhizosphere fungal community using FUNGuild 1.0 software has certain constraints. Future research will incorporate metagenomic sequencing and related technologies to validate these findings. In addition, after calculating the functional molecular ecological network, the analysis only reached the level of identifying key functions (module hubs, network hubs, and connectors) within the network, while the detailed ecological significance of peripheral nodes will be further studied in future work. Moreover, due to constraints in manpower and funding, manipulative experiments were not conducted to establish causality relationships between soil variables and fungal guilds, nor were inoculation trials or mutualism assays performed on key fungi. In future research, our team will address these issues step by step and combine genetic, demographic, or trait-based data of *A. ziyuanensis* to provide references for the rational management and ecological protection of *A. ziyuanensis* in Yinzhu Laoshan.

## 5. Conclusions

The fungal community in the rhizosphere of *A. ziyuanensis* distributed at the SBT site and the SJHT site of Yinzhu Laoshan Mountain, Guangxi, China, and the fungal community of the rhizosphere consisted of seven trophic types and 81 functional groups, all of which are dominated by symbiotic trophic fungi. Among them, the frequencies of pathological fungi of the SJHT site were significantly higher than those of the SBT site (*p* < 0.05), with significant differences (*p* < 0.05) in 11 functional groups including Plant Parasite Wood Saprotroph, Plant Saprotroph Animal Pathogen, and Arbuscular Mycorrhizal Fungi in the two study areas. An analysis of the molecular ecological network based on random matrix theory showed that the structure of the fMEN of the rhizosphere fungi of the SBT site was relatively more complex, with tighter interactions and the potential to respond faster to environmental changes, while the modularity of fungal community of the rhizosphere of the SJHT site was relatively higher, with lower average connectivity and strong resistance to external interference. Ascomycota and Basidiomycota, as important groups, play a crucial regulatory role in the community structure and function of rhizosphere fungi in *A. ziyuanensis*, while soil physicochemical factors are important factors restricting the adaptive development of fungi. This approach, which integrates community composition analysis, functional difference verification, network structure analysis, and their association with key driving factors, can reveal the adaptation mechanisms of the rhizosphere microbial communities of specific plants to their habitats. This work provides a generalizable research paradigm for understanding the synergistic relationships among endangered plants, microorganisms, and the environment, in addition to guiding the conservation of endangered plants and ecosystem restoration.

## Figures and Tables

**Table 1:** Soil physicochemical properties (mean ± standard error, *n* = 15).

Physicochemical Properties	SBT	SJHT	*p* Value	Significance
SWC (%)	40.54 ± 2.54	48.83± 4.25	0.11	-
pH	4.30 ± 0.06	4.38 ± 0.05	0.31	-
TN (g/kg)	5.57 ± 0.29	5.38 ± 0.51	0.75	-
AN (mg/kg)	285.60 ± 17.81	279.88 ± 30.69	0.87	-
NO_3_^−^-N (mg/kg)	5.01 ± 1.66	8.74 ± 3.02	0.29	-
NH_4_^+^-N (mg/kg)	31.07 ± 3.51	30.90 ± 3.37	0.97	-
SOC (g/kg)	72.71 ± 4.79	64.15 ± 4.10	0.19	-
TP (g/kg)	0.44 ± 0.08	0.71 ± 0.15	0.13	-
AP (mg/kg)	3.44 ± 0.53	4.66 ± 1.64	0.49	-
TK (g/kg)	22.27 ± 1.11	17.55 ± 1.05	0.00	**
AK (mg/kg)	95.73 ± 4.25	80.80 ± 5.32	0.04	*
MBC (mg/kg)	1062.40 ± 98.64	1092.67 ± 129.60	0.85	-
MBN (mg/kg)	456.00 ± 46.43	365.47 ± 30.31	0.12	-

Note: Site names were Shenbaotang (SBT) and Sanjiaohutang (SJHT); AK, available potassium; AN, available nitrogen; AP, available phosphorus; MBC, microbial biomass carbon; MBN, microbial biomass nitrogen; NH_4_^+^-N, ammonium nitrogen; NO_3_^−^-N, nitrate nitrogen; SOC, soil organic carbon; SWC, soil water content; TK, total potassium; TN, total nitrogen; TP, total phosphorus. - *p* > 0.05; * *p* < 0.05; ** *p* < 0.01.

**Table 2:** Relative abundance of fungal trophic modes in the rhizosphere community.

Fungal Trophic Mode	Relative Abundance (%)	
	SBT	SJHT
Symbiotroph	20.97	22.00
Saprotroph-Symbiotroph	10.61	14.16
Saprotroph	7.76	6.26
Pathotroph-Symbiotroph	2.51	2.20
Pathotroph-Saprotroph-Symbiotroph	11.17	3.96
Pathotroph-Saprotroph	5.88	8.35
Pathotroph	2.43	7.95
Total	61.33	64.88

Note: Site names were the Shenbaotang (SBT) and Sanjiaohutang sites (SJHT).

**Table 3:** Differential analysis of functional guilds in rhizospheric soil fungal communities (mean ± standard error, *n* = 15).

Fungal Functional Guilds	SBT	SJHT	*p* Value	Significance
Animal Pathogen	0.0199 ± 0.0048	0.0758 ± 0.0262	0.0327	*
Arbuscular Mycorrhizal	0.0011 ± 0.0004	0.0036 ± 0.0013	0.0136	*
Bryophyte Parasite-Dung Saprotroph-Ectomycorrhizal-Fungal Parasite-Leaf Saprotroph-Plant Parasite-Undefined Saprotroph-Wood Saprotroph	0.0146 ± 0.0087	0.0030 ± 0.0025	0.0063	**
Dung Saprotroph	0.0002 ± 0.0001	0.0003 ± 0.0003	0.0442	*
Dung Saprotroph-Plant Saprotroph-Wood Saprotroph	0.0004 ± 0.0002	0.0001 ± 0.0001	0.0231	*
Ectomycorrhizal-Lichen Parasite-Lichenized-Plant Pathogen	0.0693 ± 0.0492	0.0010 ± 0.0003	0.0000	***
Ectomycorrhizal-Undefined Saprotroph-Wood Saprotroph	0.0003 ± 0.0001	0.0027 ± 0.0019	0.0337	*
Endomycorrhizal-Plant Pathogen-Undefined Saprotroph	0.0028 ± 0.0009	0.0009 ± 0.0004	0.0284	*
NULL	0.0005 ± 0.0002	0.0002 ± 0.0001	0.0434	*
Plant Parasite-Wood Saprotroph	0.0009 ± 0.0005	0.0001 ± 0.0001	0.0044	**
Plant Saprotroph	0.0004 ± 0.0001	0.0006 ± 0.0006	0.0020	**

Note: Site names were the Shenbaotang (SBT) and Sanjiaohutang sites (SJHT). * *p* < 0.05; ** *p* < 0.01; *** *p* < 0.001.

**Table 4:** Topological parameters of the fungal functional molecular ecological networks in soils at sites SBT and SJHT.

Plot	Molecular Ecological Networks									
	Cutoff	Total Nodes	Total Links	R2 R2 of Power-Law	Number of Positive Connections	Number of Negative Connections	Average Degree (avg K)	Average Clustering Coefficient (avg CC)	Average Path Distance (GD)	Modularity Index (Module Number)
SBT	0.690	89	184	0.69	40	144	4.135	0.057	3.708	0.448 (6)
SJHT	0.690	64	115	0.74	60	55	3.594	0.169	3.391	0.524 (6)

Note: Site names were Shenbaotang (SBT) and Sanjiaohutang (SJHT).

**Table 5:** Module hubs and connectors in the fungal functional molecular ecological networks in soils at sites SBT and SJHT.

Fungal Trophic Mode	Module Hub		Connector	
	SBT	SJHT	SBT	SJHT
Symbiotroph		OTU85	OTU143, OTU103	
Pathotroph–Saprotroph–Symbiotroph			OTU298	
Saprotroph–Symbiotroph	OTU27	OTU199		OTU44, OTU117
Saprotroph			OTU92, OTU411	
Pathotroph–Saprotroph			OTU321	
Pathotroph–Symbiotroph				
Pathotroph			OTU182	

Note: OTU, operational taxonomic unit; site names were Shenbaotang (SBT) and Sanjiaohutang (SJHT); OTU143 (Lichenized), OTU103 (Ectomycorrhizal), OTU27 (Endophyte-Litter Saprotroph-Soil Saprotroph-Undefined Saprotroph), OTU92 (Wood Saprotroph), OTU411 (Plant Saprotroph-Wood Saprotroph), OTU298 (Endophyte-Plant Pathogen-Undefined Saprotroph), OTU321 (Animal Pathogen-Fungal Parasite-Undefined Saprotroph), OTU182 (Animal Pathogen), OTU85 (Endophyte), OTU44 (Ectomycorrhizal-Undefined Saprotroph), OTU117 (Ectomycorrhizal-Undefined Saprotroph), OTU199 (Endophyte-Undefined Saprotroph-Wood Saprotroph), Ascomycota (OTU85, OTU199, OTU143, OTU298, OTU92, OTU411, OTU321, OTU182, OTU117), Basidiomycota (OTU103, OTU44), Mortierellomycota (OTU27).

## Data Availability

The soil physicochemical data presented in this study are available on request from the corresponding author. All ITS rRNA gene sequencing data from this study are available in NCBI SRA under the study accession number PRJNA1241497.

## Supplementary Materials

The following supporting information can be downloaded at: https://www.mdpi.com/article/10.3390/microorganisms13091989/s1, Table S1. Sampling site information.

## Abbreviations

The following abbreviations are used in this manuscript:

FUNGuild	Fungi Functional Guild
fMEN	Functional Molecular Ecological Network
SBT	Shenbaotang
SJHT	Sanjiaohutang
