# Supplementary material for: Functional Characteristics of Fungal Communities in the Rhizosphere of the Endangered Plant Abies ziyuanensis
Source: Microorganisms. 2025 Aug 26;13(9):1989. doi: 10.3390/microorganisms13091989 (PMC12473087; doi:10.3390/microorganisms13091989)
Supplement: Supplementary file 1 [file microorganisms-13-01989-s001.zip › microorganisms-3816659-supplementary.pdf]

Supporting Tables

Table S1. Sampling site information

Number	Lon (°)	Lat (°)	Alt. (m)	DBH (cm)	H (m)	CW (m*m)	Major associated species
SBT01	26.251892	110.559908	1822	5	14	5*6	
SBT02	26.251456	110.559375	1804	37.5	19	9.5*8	
SBT03	26.251459	110.559365	1803	33.6	20	7*6	
SBT04	26.251466	110.559365	1803	28.6	18.5	8.5*5	*Fargesia spathacea, Ophiopogon*
SBT05	26.251487	110.559365	1805	27.2	18	7*4	*bodinieri, Eurya semiserrulata,*
SBT06	26.251481	110.559363	1806	27.2	18	8*6	*Illicium angustisepalum, Lyonia*
SBT07	26.251523	110.559475	1807	25.8	14	7*5	*ovalifolia, Schima superba, Quercus*
SBT08	26.251608	110.559465	1811	23.5	14	4*7	*glauca, Quercus*
SBT09	26.251672	110.559447	1816	18	13	6*5	*multinervis,Lithocarpus*
SBT 10	26.251671	110.559447	1815	20.8	12	3*5	*cleistocarpus*
SBT 11	26.251608	110.559465	1811	19.8	11	6*5	
SBT 12	26.251670	110.559447	1816	30	10	7.5*8	
SBT 13	26.251780	110.559542	1821	30.5	13	7.3*7	
SBT 14	26.253071	110.559206	1793	27.5	23	5*6.5	
SBT 15	26.253437	110.560754	1754	30	21	6*6.5	
SJHT01	26.263180	110.559345	1749	3	0.8	3*2	
SJHT02	26.263062	110.559347	1731	7	5	6*5	
SJHT03	26.262944	110.559142	1940	8	5	2.5*2	
SJHT04	26.263208	110.559326	1742	6	3.5	3.2*3	
SJHT05	26.262109	110.559377	1778	14	8	3*2.5	*Fargesia spathacea, Ophiopogon*
SJHT06	26.262181	110.559378	1783	12.3	7	3*5	*bodinieri, Viburnum fordiae,*
SJHT07	26.262275	110.559355	1785	3	2	1*1.2	*Rhododendron simsii, Rhododendron*
SJHT08	26.261343	110.558253	1773	26.8	7	6*2.5	*latoucheae, Schima superba, Quercus*

SJHT09	26.260649	110.558593	1791	4	3	1*0.9	*glauca*, *Lithocarpus cleistocarpus*
SJHT10	26.260579	110.558527	1790	12	7.5	4*3	
SJHT11	26.259697	110.556235	1839	20.5	20	6.2*5	
SJHT12	26.259493	110.556117	1843	22	17	6.4*7.3	
SJHT13	26.258370	110.555077	1871	14.2	9.5	5*5.5	
SJHT14	26.255911	110.557172	1820	10.6	7	5*6	
SJHT15	26.255900	110.557170	1821	15	9	4.3*3.2	

Note: Lon, Longitude; Lat, Latitude; Alt., Altitude; DBH, Diameter at Breast Height; H, Tree Height; CW, Crown Width.
